# Keratinocyte-Mediated Antigen Presentation in Psoriasis: Preliminary Insights from In Vitro Studies †

**DOI:** 10.3390/ijms252413387

**Published:** 2024-12-13

**Authors:** Katarzyna Zima, Dorota Purzycka-Bohdan, Aneta Szczerkowska-Dobosz, Magdalena Gabig-Cimińska

**Affiliations:** 1 Department of Medical Biology and Genetics, University of Gdansk, Wita Stwosza 59, 80-308 Gdansk, Poland; 2 Department of Physiology, Medical University of Gdansk, Debinki 1, 80-211 Gdansk, Poland; 3 Department of Dermatology, Venereology and Allergology, Medical University of Gdansk, University Clinical Centre, Mariana Smoluchowskiego 17, 80-214 Gdansk, Poland; dorota.purzycka-bohdan@gumed.edu.pl (D.P.-B.); adobosz@gumed.edu.pl (A.S.-D.); 4 Clinical Physiology Unit, Medical Simulation Centre, Medical University of Gdansk, Debowa 25, 80-204 Gdansk, Poland

**Keywords:** psoriasis, keratinocytes, autoimmune diseases, MHC class II

## Abstract

Antigen presentation plays a critical role in the pathogenesis of immune-mediated disorders. This study aimed to investigate the effects of IFN-γ and a cytokine mix (5MIX: IL-1α, IL-17A, IL-22, OsM, and TNF-α) on the antigen-presenting capabilities of keratinocytes, with a specific focus on immune-mediated dermatological conditions such as psoriasis (Ps). To achieve this, keratinocytes were treated with IFN-γ and 5MIX, and their impact on the expression of key antigen-presentation molecules, HLA-DRα and CD74, was assessed. Transcriptomic analysis revealed that IFN-γ alone altered the expression of 254 genes, highlighting its central role in modulating immune responses, including the recruitment of immune cells and regulation of inflammation. Temporal experiments further demonstrated that IFN-γ and 5MIX enhanced early endocytic activity and lysosomal degradation pathways, both essential for effective antigen presentation and T-cell activation. To extend these findings to a clinical context, a co-culture model using keratinocytes derived from psoriatic patients was established. This model revealed increased cytokine production following antigen stimulation, indicating robust and consistent CD4+ and naïve T-cell responses. These results elucidate the complex dynamics of cytokine signaling and antigen presentation in keratinocytes, providing insights into potential therapeutic strategies for immune-mediated skin disorders like Ps.

## 1. Introduction

The skin immune system (SIS) is a complex network of effector cells and molecular mediators pivotal for orchestrating innate and adaptive immunological responses. It encompasses a diverse array of immune cells, including dendritic cells (DCs), Langerhans cells (LCs), T lymphocytes, and antigen-presenting cells (APCs) [1]. Keratinocytes (KCs), the prevalent cell type within the epidermis, have been shown to express Major Histocompatibility Complex class I (MHC I) molecules constitutively. When activated, they can function as non-professional APCs due to their induction of MHC II expression under inflammatory conditions, including interferon gamma (IFN-γ) stimulation [2]. However, the low expression of co-stimulatory molecules CD80 and CD86 has led to debates regarding the ability of keratinocytes to stimulate T cells, with some suggesting that they may induce anergy and tolerance instead [3]. However, although lacking CD80/CD86, KCs induce a metabolic change in T cells. As a result, KCs presumably regulate T cell activities in the skin via two different co-stimulatory receptors: CD2 and CD6 [4]. Under proinflammatory conditions, primary human KCs can directly activate naïve human T cells through cell contact and co-stimulatory signals, specifically via CD58/CD2 and CD54/LFA-1 pathways [5]. 

The expression of MHC II on skin cells, such as melanocytes, is noted in inflammatory skin diseases and certain tumor cases, such as melanoma, highlighting the relevance of this phenomenon in pathological conditions [6]. Moreover, the unexpected role of MHC II expression by keratinocytes in controlling homeostatic type 1 responses to the microbiota underscores the significance of keratinocytes as APCs in maintaining skin immune homeostasis [7]. In the context of psoriasis (Ps), CD1a+ LCs (Human Leukocyte Antigen – DR isotype, HLA-DR+), CD1a–DCs (HLA-DR+), and KCs (HLA-DR+), are all APCs that contribute to the pathogenesis of the disease [8]. KCs effectively present antigens to memory CD4+ and CD8+ T lymphocytes via MHC II and ICAM-1, activated by IFN-γ, triggering functional responses [3]. Commensal microbes also promote MHC II expression in KCs, reinforcing their APC role [9]. Moreover, the involvement of keratinocytes in antigen presentation has been demonstrated in the context of viral infections. Keratinocytes have been shown to present the herpes simplex virus antigen directly to T lymphocytes, suggesting an accessory antigen-presenting role in herpetic skin lesions [10]. Similarly, the human papillomavirus E7 protein has been found to inhibit the IFN-γ enhancement of keratinocyte antigen processing and T-cell lysis, indicating the complex interplay between viral proteins and keratinocyte antigen presentation [11]. Beyond their role as non-professional APCs, KCs significantly contribute to the immune response through cytokine and chemokine production [12]. In summary, the multifaceted role of KCs in immune regulation, antigen presentation, and cytokine modulation release underscores their significance in the skin immune system. Understanding the intricate interplay between KCs and immune cells, particularly in the context of psoriasis, is vital for understanding skin disease mechanisms and identifying new treatment avenues. This study aims to investigate the modulatory effects of IFN-γ and a proinflammatory cytokine mix (5MIX: IL-1α, IL-17A, IL-22, OsM, and TNF-α) on keratinocyte antigen-presenting capacities and their interactions with CD4+ lymphocytes, with a specific focus on their role in psoriasis pathogenesis. The investigation explores how these cytokines regulate key antigen-presentation molecules, such as HLA-DRα and CD74, in keratinocytes, alongside their impact on antigen processing dynamics, including the colocalization of antigens with endosomal and lysosomal markers. Furthermore, transcriptomic profiling reveals distinct patterns of gene expression induced by IFN-γ, 5MIX, and their combination, shedding light on the activation of specific immune pathways and transcriptional regulators in keratinocytes. Finally, the functional consequences of these cytokine treatments are assessed using co-culture models of keratinocytes with CD4+ and naïve CD4+ lymphocytes, elucidating cytokine production patterns in untreated, topically treated, and biologically treated psoriatic patients. These findings offer valuable insights into the immune regulatory roles of keratinocytes in psoriasis and highlight potential therapeutic targets for modulating inflammatory responses in immune-mediated skin disorders.

## 2. Results

### 2.1. Increased HLA-DRα and CD74 Levels in a 5MIX and/or IFN-γ Treatment In Vitro 2D Normal Human Epidermal Keratinocytes (NHEK) Model

**Figure 1:**
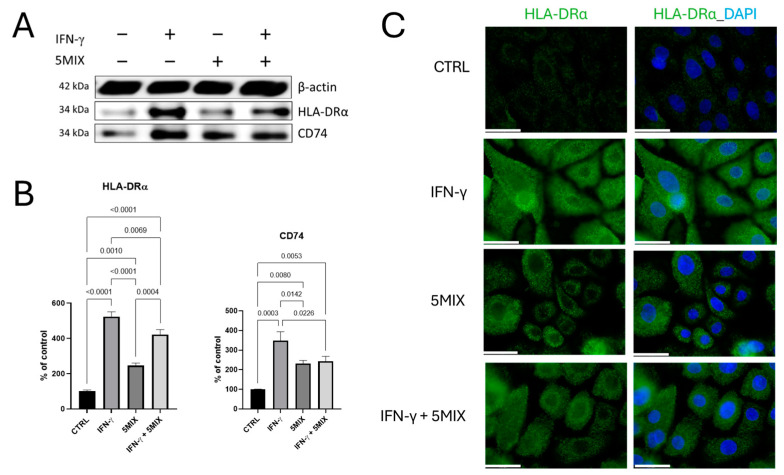
Elevation of HLA-DRα and CD74 expression in IFN-γ stimulated Normal Human Epidermal Keratinocytes (NHEKs) 2D in vitro model. The effect of interferon gamma (IFN-γ; 500 U/mL); 5MIX: interleukin (IL)-1α, IL-17A, IL-22, oncostatin M (OsM), and tumor necrosis factor alpha (TNF-α), at 2 ng/mL each; and the combination of 5MIX (2 ng/mL each) with IFN-γ (500 U/mL) on the levels of HLA-DRα and CD74 (**A**) proteins, in an in vitro 2D model of NHEK. The graphs represent the results of densitometric analyses normalized to β-actin, presented as a percentage of the control, non-treated cells (**B**). Representative immunofluorescence images (**C**) show the subcellular localization of HLA-DRα (green) with DAPI-stained nuclei (blue). Ten microscopic fields, each comprising 50–100 cells, were arbitrarily chosen for imaging. The scale bar equals 35.6 µm. All experiments were conducted in biological triplicates (*n* = 3), and comparisons with *p* < 0.05 are displayed.

To quantify the effects of interferon gamma (IFN-γ) (500 U/mL); a cytokine mix comprising interleukin (IL)-1α, IL-17A, IL-22, oncostatin M (OsM), and tumor necrosis factor alpha (TNF-α) (5MIX, 2 ng/mL each); and their combination (5MIX + IFN-γ) on the levels of Human Leukocyte Antigens (HLAs) class II histocompatibility antigen, DR alpha chain (HLA-DRα), and Cluster of Differentiation 74 (CD74) proteins in Normal Human Epidermal Keratinocytes (NHEKs), immunoblotting was utilized (Figure 1A,B). The basal levels of both HLA-DRα and CD74 proteins were determined in untreated (control) KCs, which served as a baseline for comparative analysis. Treatment with IFN-γ alone resulted in the most significant upregulation of HLA-DRα and CD74 protein levels. Densitometry analysis revealed that the expression of HLA-DRα and CD74 proteins was substantially elevated compared to the control, with HLA-DRα exhibiting a 5.2-fold increase (*p* < 0.0001) and CD74 showing a 3.5-fold increase (*p* = 0.0003). Keratinocytes treated with the 5MIX demonstrated elevated expression of HLA-DRα and CD74, though to a lesser extent than observed with IFN-γ treatment. Densitometric analysis quantified a 0.5-fold increase in HLA-DRα (*p* = 0.001) and a 0.7-fold increase in CD74 (*p* = 0.008) protein levels compared to the control group. The combined treatment with IFN-γ and 5MIX resulted in a synergistic elevation in HLA-DRα and CD74 protein levels, albeit slightly less than seen with IFN-γ treatment alone. Densitometry revealed a 1.7-fold increase for HLA-DRα (*p* < 0.0001) and a 1.1-fold increase in CD74 (*p* = 0.0053) compared to the control (Figure 1A,B). Additionally, an immunofluorescence analysis of HLA-DRα in NHEKs confirmed the results observed with immunoblotting. Fluorescent microscopy revealed an increased HLA-DRα expression localized within the cells following IFN-γ treatment, with a slightly less pronounced increase observed for the 5MIX and combined treatments (Figure 1C). These findings corroborate the densitometry data, further highlighting the significant upregulation of HLA-DRα under IFN-γ stimulation, with modest increases under 5MIX and combined treatment conditions (Figure 1C).

### 2.2. IFN-γ, Both Alone and in Combination with 5MIX, Significantly Enhances the Colocalization of OVA with Early Endosomal and Lysosomal Markers in NHEKs in a Time-Dependent Manner

**Figure 2:**
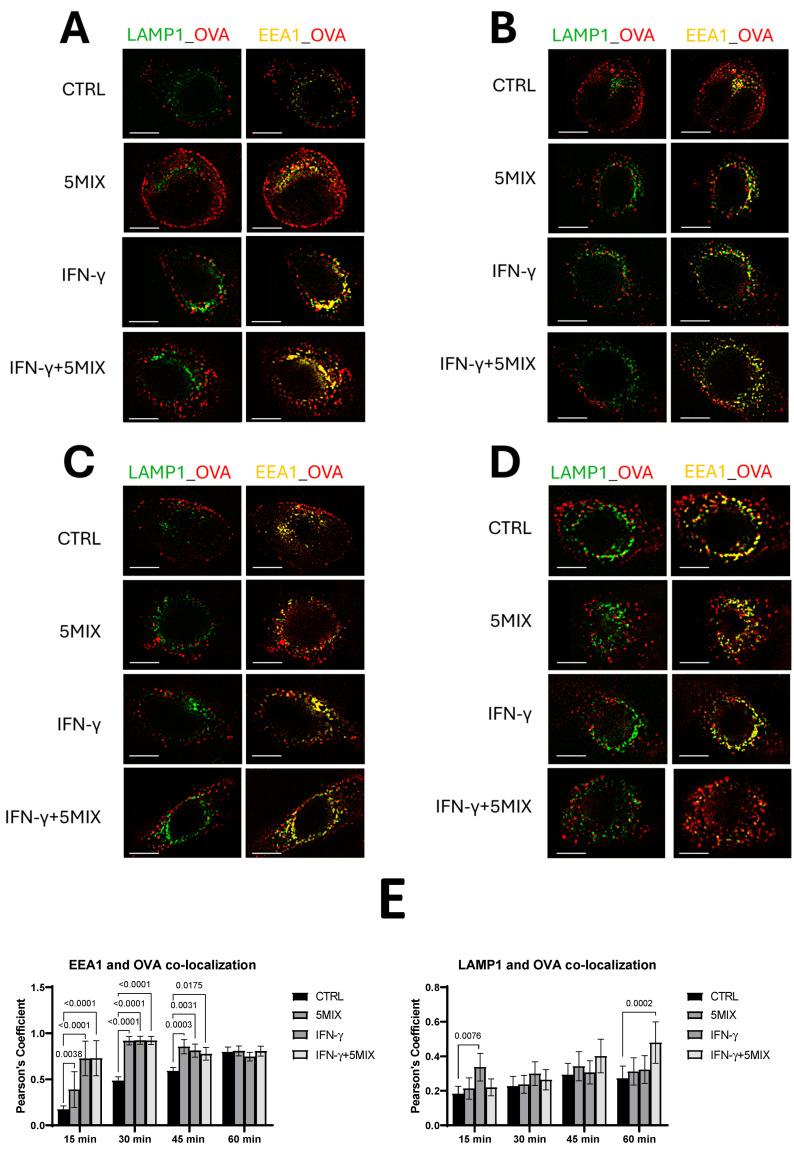
Synergistic enhancement of ovalbumin (OVA, used as a model antigen) internalization, through colocalization with early endosomal and lysosomal markers was analyzed in Normal Human Epidermal Keratinocytes (NHEKs) treated with interferon gamma (IFN-γ; 500 U/mL) and/or 5MIX, a cytokine mix comprising interleukin (IL)-1α, IL-17A, IL-22, oncostatin M (OsM), and tumor necrosis factor alpha (TNF-α), each at 2 ng/mL, in a time-dependent study. Ten microscopic fields, each containing 50–100 cells, were arbitrarily chosen for each of the three separate experiments (*n* = 3), and representative images were captured. The scale bar equals 10 µm. The colocalization of OVA with the early endosome marker 1 (EEA1) and lysosomal-associated membrane protein 1 (LAMP1) was evaluated at four time points: 15 min (**A**), 30 min (**B**), 45 min (**C**), and 60 min (**D**) using an in vitro 2D NHEK model. Quantification of colocalization levels was performed using Pearson’s correlation coefficient (**E**). All experiments were conducted in biological triplicates (*n* = 3), and comparisons with *p* < 0.05 are indicated.

Antigen processing is a crucial step in the immune response, involving the uptake of antigens via endocytosis and their subsequent trafficking through intracellular compartments for processing and presentation on major histocompatibility complex (MHC) class II molecules. This process is essential for activating CD4+ T cells. In this study, we utilized immunofluorescence microscopy to investigate the colocalization of ovalbumin (OVA), a model antigen, with markers of intracellular trafficking pathways, specifically Early Endosome Antigen 1 (EEA1) and Lysosomal-Associated Membrane Protein 1 (LAMP1). These markers indicate early endosomes and lysosomes, respectively, and provide insight into the dynamics of antigen uptake and routing within keratinocytes. NHEKs were treated with 5MIX, IFN-γ, or their combination to evaluate how these inflammatory stimuli influence antigen processing and the capacity of keratinocytes to function as non-professional antigen-presenting cells. The temporal dynamics of this colocalization were scrutinized at intervals of 15 min (Figure 2A), 30 min (Figure 2B), 45 min (Figure 2C), and 60 min (Figure 2D) post-incubation (Figure 2). A quantitative analysis of colocalization was conducted through the computation of Pearson’s correlation coefficient (Figure 2E).

At the 15 min mark, colocalization metrics already delineated a distinct uptick in OVA-EEA1 overlap within cultures treated with IFN-γ alone (*p* < 0.0001) and in combination with 5MIX (*p* < 0.0001), compared to the control group. The 5MIX treatment alone also showed enhanced colocalization, though to a lesser extent (*p* < 0.0001). This trend persisted at the 30 and 45 min intervals, with significantly higher levels of OVA-EEA1 colocalization levels being maintained by the 5MIX (*p* < 0.0001 at 30 min and *p* = 0.0003 at 45 min), IFN-γ (*p* < 0.0001 at 30 min and *p* = 0.0031 at 45 min), and the combination of IFN-γ with 5MIX (*p* < 0.0001 at 30 min and *p* = 0.0175 at 45 min) treatments. Such enhancement, relative to the control, highlights a continued preferential engagement with the early endosomal machinery, likely indicative of an enhanced antigen processing capacity triggered by cytokine signaling. Interestingly, by the 60 min time point, the colocalization levels across all conditions—including control, IFN-γ, 5MIX, and 5MIX + IFN-γ—converged, equalizing to analogous magnitudes. This equilibration insinuates that, notwithstanding initial differences in antigen uptake and processing velocities, the terminal routing and processing of OVA within keratinocytes reach a steady state across all treatment conditions by the one-hour threshold (Figure 2B). A notable increase in OVA and LAMP1 colocalization was observed in keratinocytes treated with IFN-γ alone (*p* = 0.0076), indicating a rapid initiation of antigen processing towards lysosomal degradation. At intermediate checkpoints (30 and 45 min), no significant differences in OVA-LAMP1 colocalization levels were observed across any treatment groups. Distinctively, at the 60 min mark, a significant increase in OVA-LAMP1 colocalization was observed in cultures treated with the combination of 5MIX+ IFN-γ (*p* = 0.0002) (Figure 2A,B). The findings reveal that IFN-γ, both alone and in combination with 5MIX, markedly augments antigen processing in keratinocytes, as evidenced by increased colocalization with endosomal and lysosomal markers, which stabilizes across all conditions within an hour, indicating a time-dependent immune response modulation.

### 2.3. Transcriptomic Profiling of Keratinocytes Reveals Most Differential Expression Patterns in Response to IFN-γ Treatment 

**Figure 3:**
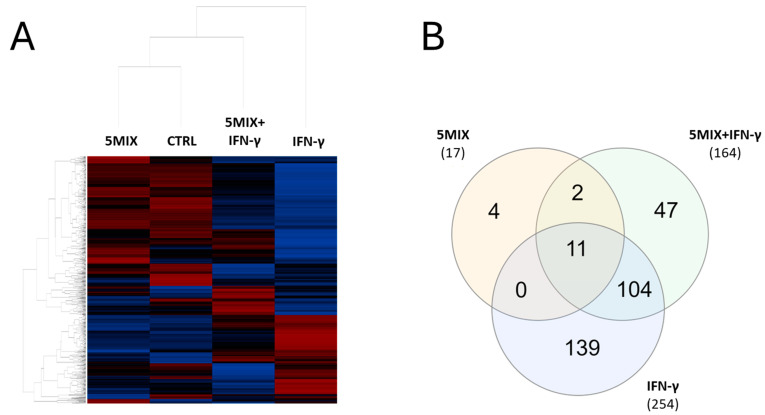
The transcriptomic landscape of Normal Human Epidermal Keratinocytes (NHEKs) unveils differential expression paradigms in response to 5MIX (interleukin (IL)-1α, IL-17A, IL-22, oncostatin M (OsM), and tumor necrosis factor alpha (TNF-α), at 2 ng/mL each) and/or interferon gamma (IFN-γ) at 500 U/mL stimulation. A heatmap visualization of gene expression data from NHEKs treated with 5MIX; IFN-γ at 500 U/mL; and a combination of 5MIX and IFN-γ (**A**). The intensity of color reflects the level of gene expression: deep red represents higher expression levels, while dark blue signifies lower expression levels. Panel (**B**) illustrates a Venn diagram comparing the number of differentially expressed genes (DEGs) across the three experimental conditions: 5MIX, IFN-γ, and 5MIX + IFN-γ. The numbers in each section represent the count of unique or overlapping DEGs in the different treatment groups. IFN-γ treatment resulted in 254 DEGs, 5MIX in 17 DEGs, and the combination of 5MIX + IFN-γ modulated 164 DEGs. Overlapping regions indicate genes shared among the conditions. The Venn diagram was generated using the InteractiVenn web-based tool [13]. All experiments were conducted in biological triplicates (*n* = 3), and comparisons with *p* < 0.05 are displayed.

The dynamic interaction between cytokines and keratinocytes plays a pivotal role in modulating immune responses in inflammatory skin conditions such as psoriasis. To better understand these interactions at a molecular level, transcriptomic profiling provides a powerful tool for identifying differentially expressed genes and uncovering key pathways that govern keratinocyte function under various cytokine treatments. In this study, we employed the NanoString^®^ nCounter^®^ system to perform comprehensive transcriptional profiling of keratinocytes treated with IFN-γ, 5MIX, and their combination. This analysis revealed distinct gene expression patterns and highlighted critical pathways involved in antigen presentation, immune signaling, and inflammatory modulation. The comprehensive transcriptional profiling of keratinocytes using the NanoString^®^ nCounter^®^ system identified distinct patterns of gene expression elicited by 5MIX, IFN-γ, and 5MIX + IFN-γ in combination (Figure 3A). Statistically significant changes were observed only in 17 genes after 5MIX treatment, while IFN-γ alone influenced 254 genes (Figure 3B). The combined stimulation altered the expression of 164 genes, suggesting a complex interplay between these cytokines in modulating keratinocyte responses (Figure 3B). The Venn diagram (Figure 3B) reveals that the highest overlap occurs between the IFN-γ and 5MIX + IFN-γ conditions, with 104 shared genes, highlighting the dominant influence of IFN-γ in the combined treatment. In contrast, there are no shared genes between the 5MIX and IFN-γ conditions, indicating distinct regulatory effects when these treatments are applied individually (Figure 3B). 

**Figure 4:**
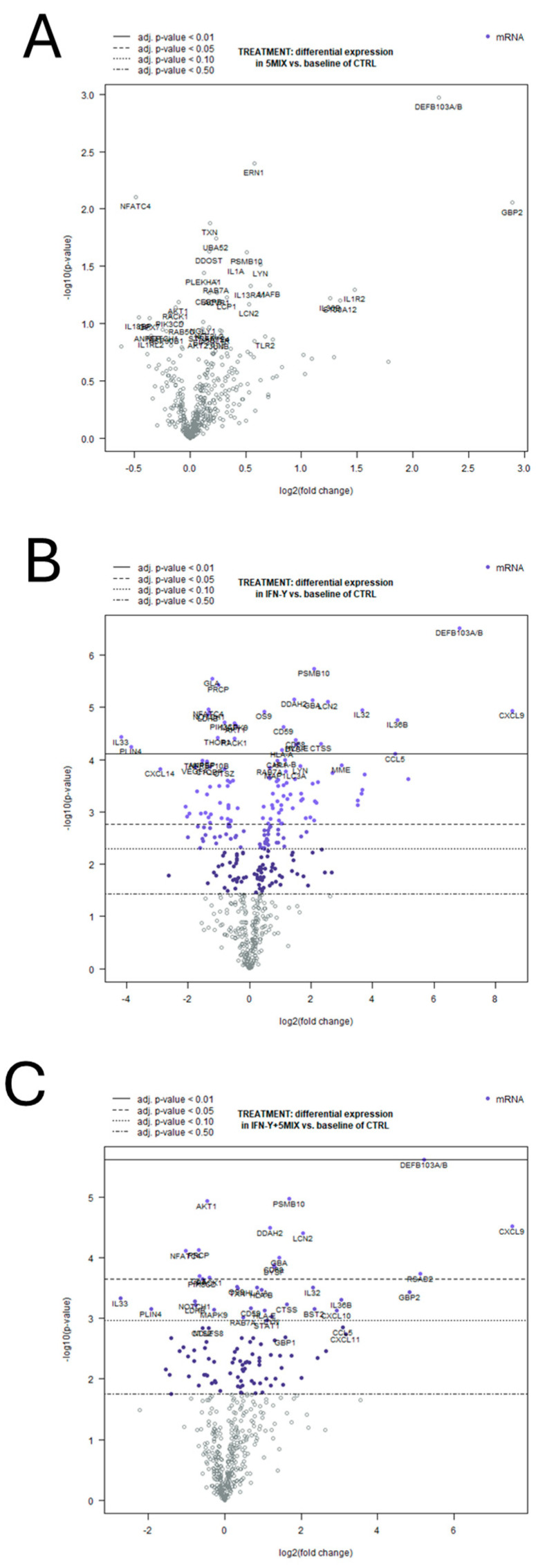
Differential gene expression in Normal Human Epidermal Keratinocytes (NHEKs) in response to 5MIX (interleukin (IL)-1α, IL-17A, IL-22, oncostatin M (OsM), and tumor necrosis factor alpha (TNF-α), at 2 ng/mL each) and/or interferon gamma (IFN-γ) at 500 U/mL stimulation. Volcano plots illustrate the statistical significance versus the magnitude of gene expression changes in NHEKs upon treatment with 5MIX (**A**), IFN-γ (**B**), and their combination (**C**). Each point on the plots represents a gene, with the *x*-axis displaying the log2 fold change and the y-axis showing the negative log10 of the adjusted *p*-value. Genes with statistically significant differential expression are highlighted in blue and annotated with their respective gene symbols. The horizontal dashed lines represent thresholds for adjusted *p*-values: *p* < 0.01, *p* < 0.05, and *p* < 0.10. These thresholds demarcate varying levels of statistical significance, with genes above the dashed lines meeting the respective significance criteria. All experiments were conducted in biological triplicates (*n* = 3).

**Figure 5:**
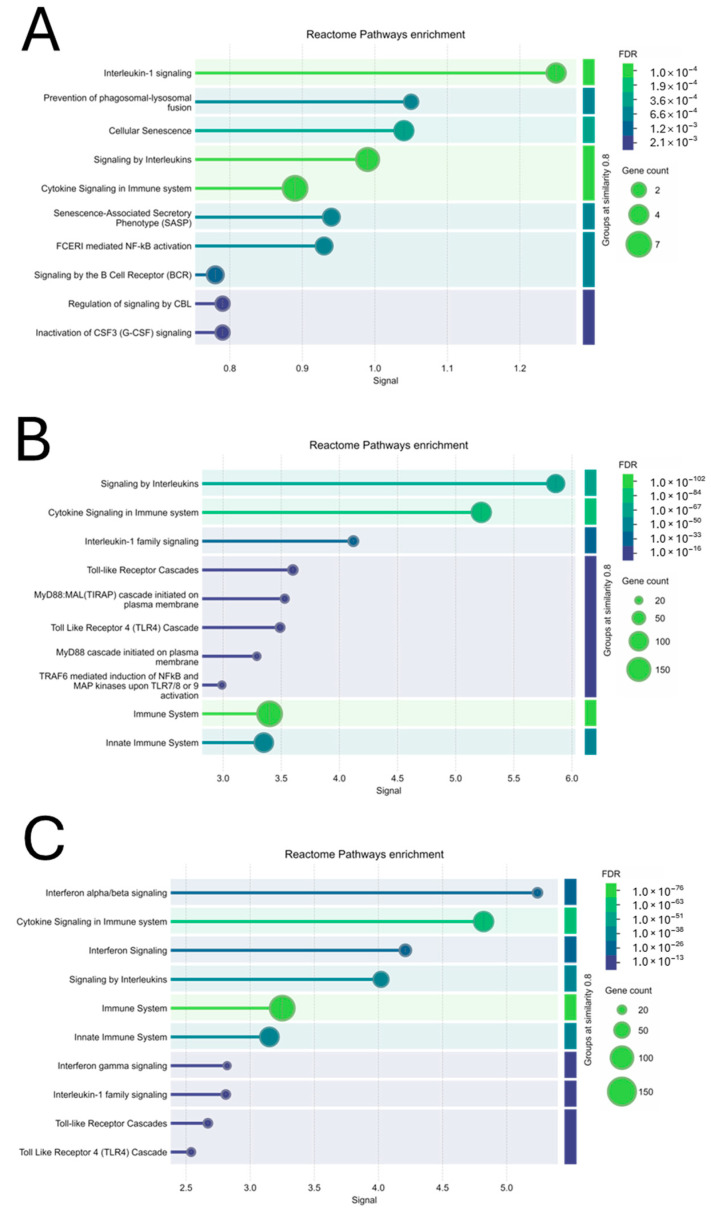
Pathway enrichment analysis of differentially expressed genes (DEGs) in Normal Human Epidermal Keratinocytes (NHEKs) treated with 5MIX (interleukin (IL)-1α, IL-17A, IL-22, oncostatin M (OsM), and tumor necrosis factor alpha (TNF-α), at 2 ng/mL each) and/or interferon gamma (IFN-γ) at 500 U/mL. Bubble plots illustrate Reactome pathway enrichment for DEGs in NHEKs upon treatment with 5MIX (**A**), IFN-γ (**B**), and their combination (**C**), compared to the control (CTRL). The x-axis shows the Reactome pathways, while the y-axis represents the significance level of enrichment (−log10 of the false discovery rate (FDR)). Bubble sizes correspond to the number of genes associated with each pathway, while the color gradient indicates FDR values, with darker blue representing higher statistical significance and lighter green reflecting less significant enrichment. All analyses were conducted using the STRING platform (https://string-db.org/; accessed on 23 November 2024) [14], and all experiments were performed in biological triplicates (*n* = 3).

In the 5MIX-treated keratinocytes, a limited number of genes exhibited significant differential expression compared to the control (Figure 4A). Among these, *DEFB103A/B* and *GBP2* showed notable upregulation, with log2 fold changes of 2.23 (*p* = 0.00107) and 2.89 (*p* = 0.00884), respectively, suggesting a strong activation of innate immune responses (Table 1A). In contrast, *NFATC4* was downregulated with a log2 fold change of −0.485 (*p* = 0.00786), indicating the potential modulation of transcriptional activity linked to immune regulation or keratinocyte differentiation (Table 1A). Pathway analysis further illuminated the biological implications of 5MIX treatment, highlighting the most significantly altered pathways based on the signal score, which integrates the strength of enrichment and the false discovery rate. The pathway Interleukin-1 signaling (signal = 1.25) emerged as the most prominent, reflecting its central role in driving inflammatory responses (Figure 5A). Similarly, signaling by Interleukins (signal = 0.99) and Cellular Senescence (signal = 1.04) highlighted the activation of cytokine-related pathways and stress responses (Figure 5A). Notably, the prevention of phagosomal–lysosomal fusion (signal = 1.05) suggested disruptions in cellular degradation processes (Figure 5A). 

The response to IFN-γ alone was particularly pronounced, with notable upregulation in genes associated with immune responses. It is noteworthy that, among the significantly modulated genes, *CXCL9* emerged as the most upregulated entity, exhibiting an extraordinary log2 fold change of 8.54 (*p* = 1.18 × 10^−5^), indicative of IFN-γ’s potent effect on chemokine expression, potentially modulating immune cell recruitment and inflammatory responses. Similarly, *DEFB103A/B* and *IL36B* were significantly upregulated, with log2 fold changes of 6.82 (*p* = 3.08 × 10^−7^) and 4.82 (*p* = 1.73 × 10^−5^), respectively, highlighting the cytokine’s role in enhancing antimicrobial defense mechanisms and pro-inflammatory signaling within the epidermal compartment (Table 1B). Conversely, the analysis revealed the significant downregulation of critical genes, with *IL33* showing the most pronounced decrease, a log2 fold change of −4.18 (*p* = 3.73 × 10^−5^). The downregulation of *GLA* and *PRCP*, with log2 fold changes of −1.2 (*p* = 2.82 × 10^−6^) and −1.01 (*p* = 3.67 × 10^−6^), respectively, further delineates IFN-γ’s multifaceted regulatory effects (Table 1B). Next, transcriptomic analysis identified significant pathway activations in keratinocytes treated with IFN-γ. Based on the signal score, the pathway Signaling by Interleukins (signal = 5.86) emerged as the most significantly activated, indicating robust activation of cytokine signaling (Figure 5B). Similarly, Cytokine Signaling in the Immune System (signal = 5.22) and Interleukin-1 Family Signaling (signal = 4.12) highlighted IFN-γ’s role in orchestrating immune and inflammatory responses (Figure 5B). Pathways such as Toll-like Receptor Cascades (signal = 3.6) and MyD88-dependent cascades (signal = 3.53) revealed the activation of innate immune mechanisms (Figure 5B). 

Keratinocytes exposed to the combined treatment of 5MIX and IFN-γ exhibited a wide array of transcriptional modifications. *DEFB103A/B* and *CXCL9* showed substantial upregulation with log2 fold changes of 5.22 (*p* = 2.44 × 10^−6^) and 7.51 (*p* = 3.07 × 10^−5^), respectively. Conversely, *NFATC4* and *PRCP* were significantly downregulated, with log2 fold changes of −1.02 (*p* = 7.76 × 10^−5^) and −0.672 (*p* = 7.53 × 10^−5^), respectively. This suggests the modulation of signaling pathways involved in immune responses and protease activity. Other genes like *LCN2*, *DDAH2*, and *GBA* displayed upregulation, with log2 fold changes of 2.04 (*p* = 3.96 × 10^−5^), 1.18 (*p* = 3.22 × 10^−5^), and 1.43 (*p* = 9.89 × 10^−5^), indicating roles in lipid metabolism, nitric oxide signaling, and lysosomal function. *IL32* and HLA genes (*HLAA* and *HLAB*) showed elevated expression, highlighting the enhancement of cytokine signaling and antigen presentation (Table 1C). For keratinocytes treated with the combination of IFN-γ and 5MIX, transcriptomic analysis revealed significant pathway activations. The Interferon Alpha/Beta Signaling pathway exhibited the highest signal score (5.24), indicating strong involvement of interferon-related responses (Figure 5C). Additionally, Cytokine Signaling in the Immune System (signal = 4.82) and Interferon Signaling (signal = 4.21) were prominently enriched, reflecting the treatments’ influence on key immune regulatory networks (Figure 5C). Other pathways, such as Signaling by Interleukins (signal = 4.02) and Toll-like Receptor Cascades (signal = 2.67), point to the modulation of both inflammatory and immune-related processes by combined stimulation (Figure 5C).

### 2.4. Naïve CD4+ and CD4+ Lymphocytes Exhibit Enhanced Cytokine Production Following Antigen Presentation by 5MIX and/or IFN-γ-Treated NHEKs 

**Figure 6:**
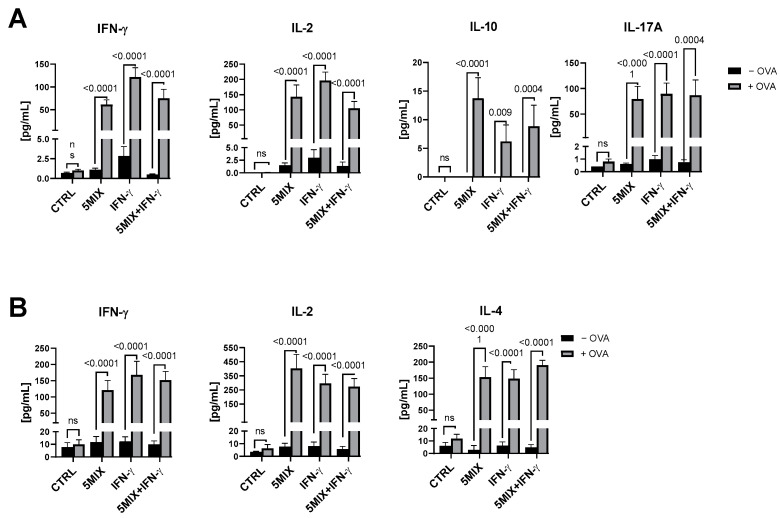
Treatment with 5MIX (IL-1α, IL-17A, IL-22, oncostatin M (OsM), and TNF-α at 2 ng/mL each) and/or IFN-γ at 500 U/mL, in combination with ovalbumin (OVA), significantly modulates cytokine production in naïve CD4+ and CD4+ lymphocytes. The levels of IFN-γ, IL-2, IL-10, and IL-17A cytokines in the supernatants obtained from a co-culture of Normal Human Epidermal Keratinocytes (NHEKs) and normal CD4+ lymphocytes (**A**), and IL-2, IL-10, and IL-17A in the supernatants from a co-culture of keratinocytes and normal naïve CD4+ lymphocytes (**B**). NHEKs were stimulated with a combination of 5MIX; IFN-γ; and the combination of 5MIX with IFN-γ for 24 h. Subsequently, OVA at 100 µg/mL was introduced as the antigen. After 24 h, the cultures were washed, and CD4+ lymphocytes were added for an additional 24 h. The quantification of cytokine production was performed using Luminex^®^ assays. All experiments were conducted in biological triplicates (*n* = 3), and comparisons with *p* < 0.05 are displayed.

The co-culture model employed in this study facilitated an examination of keratinocyte and CD4+ lymphocyte interactions in response to combined cytokine stimulations and antigen exposure. By scrutinizing in vitro cultures with and without ovalbumin, the study aimed to understand the impact of antigenic stimulation on cytokine production. Keratinocytes were stimulated with 5MIX, IFN-γ, or their combination, followed by the incorporation of OVA as a model antigen. Subsequently, washing procedures were instituted to excise any lingering stimuli prior to the admission of CD4+ or naïve CD4+ T cells into the keratinocyte cultures. Cytokine levels were quantified using the sensitive Luminex^®^ assay platform in the supernatants derived from CD4+ T cell cultures (Figure 6A) and from naïve CD4+ T cell cultures (Figure 6B).

IFN-γ, a pivotal immunoregulatory cytokine, was substantially elevated in samples with added OVA across the 5MIX, IFN-γ, and 5MIX + IFN-γ groups, with the differences achieving substantial statistical significance (*p* < 0.0001). Stimulation with IFN-γ, 5MIX, or their amalgamation precipitated a notable augmentation of IL-2 in the OVA-present conditions, yielding *p*-values < 0.0001 for these groups. A congruent trend was observed with IL-10, where its levels were significantly enhanced in the + OVA condition within the 5MIX and 5MIX + IFN-γ clusters (*p* < 0.0001 and *p* = 0.009, respectively). The ensemble subjected solely to IFN-γ also showed an appreciable increase in IL-10 levels (*p* = 0.0004). The expression profile of IL-17A paralleled the patterns observed with IFN-γ, with the introduction of OVA significantly amplifying IL-17A output subsequent to preliminary engagements with 5MIX (*p* < 0.0001) and the integrated 5MIX and IFN-γ stimulus (*p* = 0.0004). The untreated control cultures exhibited no detectable response to OVA incorporation, maintaining their basal cytokine profiles irrespective of OVA presence (Figure 6A).

Upon encountering keratinocytes treated with IFN-γ, 5MIX, or the synergistic combination in the presence of OVA, naïve CD4+ T cells exhibited profound modulations in IFN-γ output (*p* < 0.0001). Interaction with keratinocytes subjected to IFN-γ and 5MIX treatments, both singly and collectively, precipitated a statistically significant enhancement in IL-2 secretion by naïve T cells in the presence of OVA, with *p*-values < 0.0001. Concomitantly, IL-4 expression demonstrated analogous escalation, with notable upregulation in naïve T cells co-cultured with keratinocytes that had undergone pretreatment with IFN-γ, 5MIX, and the 5MIX + IFN-γ ensemble in the OVA milieu (*p* < 0.0001) (Figure 6B). The augmented production of IFN-γ, IL-2, and IL-4 by naïve lymphocytes suggests a potential shift towards a coordinated Th1 and Th2 immune response. The production of cytokines may signify an ongoing immune reaction, pertinent in contexts such as infection or autoimmune diseases. Importantly, control groups displayed no significant changes in cytokine levels with or without OVA (ns), underscoring the pivotal role of cytokine-induced keratinocyte signals in initiating and modulating naïve T cell responses (Figure 6B).

### 2.5. Response of Naïve CD4+ and CD4+ Lymphocytes Is Augmented Following Antigen Presentation by Autologous Psoriatic Human Keratinocytes

**Figure 7:**
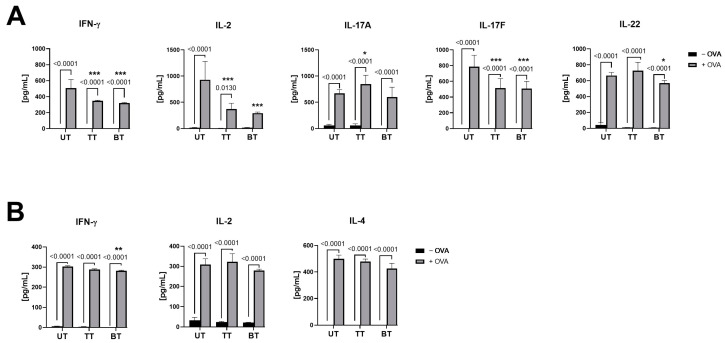
Biological interventions exhibit the modulation of immunological responses in psoriasis compared to the control, as evidenced by slightly reduced cytokine production following antigen stimulation. The levels of interferon gamma (IFN-γ), interleukin (IL)-2, IL-17A, IL-17F, and IL-22 in the supernatants from a co-culture of Psoriatic Human Epidermal Keratinocytes (PHEKs) and CD4+ lymphocytes (**A**), and IL-2, IL-10, and IL-17A in the supernatants from a co-culture of PHEKs and naïve CD4+ lymphocytes (**B**), measured from autologous, untreated psoriatic patients (UT), those subjected to topical treatment (TT), and those receiving biological treatment (BT). The keratinocytes were stimulated with IFN-γ (500 U/mL) for 24 h. Subsequently, ovalbumin (OVA) at 100 µg/mL was introduced as the antigen. After 24 h, the cultures were washed, and CD4+ lymphocytes were added for an additional 24 h. Cytokine production quantification was carried out using Luminex^®^ assays. Comparisons yielding *p*-values less than 0.05 are presented. Statistical significance between groups (UT vs. TT and UT vs. BT) is indicated with asterisks (* *p* < 0.05, ** *p* < 0.01, *** *p* < 0.001). Numerical *p*-values are provided for comparisons within each group (before and after OVA stimulation). All experiments were conducted in biological replicates (*n* = 13), with *n* = 5 for UT, *n* = 4 for TT, and *n* = 4 for BT.

Cytokine secretion profiles were quantified in co-cultures comprising autologous psoriatic keratinocytes (PHEKs) and lymphocytes to evaluate the immunological effects of patient treatment regimens for psoriasis. The cohorts included five psoriatic patients who were untreated (UT), four patients undergoing topical treatment (TT), and four patients receiving biological treatment (BT). All patients were characterized by assessments of disease severity through the Psoriasis Area and Severity Index (PASI), Body Surface Area (BSA), and the Dermatology Life Quality Index (DLQI) (Table 2). Upon IFN-γ stimulation, PHEKs adopted an activated phenotype conducive to proficient antigen presentation. The subsequent introduction of OVA for a duration of 24 h was designated to emulate an antigenic challenge, thus enabling an evaluation of immune response specificity within the keratinocyte-CD4+ lymphocyte co-cultures (Figure 7A). In the absence of OVA, cytokine levels within all psoriatic patient groups remained unchanged, without any evidence of elevation. Conversely, the incorporation of OVA elicited an uptick in cytokine production, signaling a targeted response towards the antigen. In co-cultures derived from UT patients in the presence of OVA, the concentrations of IFN-γ, IL-2, IL-17A, IL-17F, and IL-22 markedly exceeded their respective −OVA controls, with *p* < 0.0001, underscoring the immunological excitation induced by OVA. For TT patients, cytokine responses to OVA (+OVA) were similarly elevated in contrast to the −OVA condition (*p* < 0.0001, *p* = 0.013, *p* < 0.0001, *p* < 0.0001, and *p* < 0.0001 for IFN-γ, IL-2, IL-17A, IL-17F, and IL-22, respectively). Notably, no significant alterations in IL-2 levels were discerned post-OVA stimulation. Furthermore, while an increase in IFN-γ, IL-17A, IL-17F, and IL-22 production (*p* < 0.0001 for each) was observed subsequent to OVA stimulation (+OVA), these levels were comparatively reduced compared to the untreated (UT) cohort (Figure 7A), insinuating that biological treatments may attenuate the typically robust immune response characteristic of psoriatic pathology. The comparison of cytokine profiles in the co-culture of keratinocytes and CD4+ lymphocytes revealed distinct effects of topical treatment (TT) and biological treatment (BT) after OVA stimulation (Figure 7A). In the TT group, cytokine profiles showed significant differences compared to untreated psoriatic patients (UT), with reductions in IFN-γ (*p* = 0.0002), IL-2 (*p* = 0.0001), and IL-17F (*p* = 0.0005), but slight increases in IL-17A (*p* = 0.0490). The BT group demonstrated a more profound modulation of cytokine responses, characterized by significant reductions in IFN-γ (*p* < 0.0001), IL-2 (*p* < 0.0001), IL-17F (*p* = 0.0004), and IL-22 (*p* = 0.0224) compared to UT (Figure 7A).

Employing a parallel experimental arrangement with naïve CD4+ lymphocytes extracted from identical psoriatic patient groups unveiled similar patterns. Basal cytokine outputs in the absence of OVA were minimal. Following OVA induction, a surge in cytokine levels was observable across all patient groups, with cytokine production being congruent across the board, each comparative metric attaining statistical significance at *p* < 0.0001 (Figure 7B).

## 3. Discussion 

Our investigation elucidated that interferon-gamma (IFN-γ) significantly enhances the antigen-presenting capabilities of keratinocytes, underscoring the cytokine’s pivotal role in modulating immune responses within the skin. This augmentation by IFN-γ is critical for initiating effective adaptive immune responses, especially pertinent in the milieu of inflammatory skin conditions such as psoriasis (Ps). The observed enhancement in the expression levels of HLA-DRα and CD74 in keratinocytes following treatment with IFN-γ, alongside a 5MIX cytokine mix, initiates an elevated state of immunological vigilance. The pronounced upregulation of antigen presentation machinery by IFN-γ is consistent with its well-documented function in immunological activation, thus amplifying the cytokine’s therapeutic viability in the treatment of psoriatic lesions where immunological tolerance may be abrogated [15]. Intriguingly, although the concomitant application of IFN-γ with the 5MIX cytokine ensemble did not exceed the efficacy of IFN-γ alone, it precipitated a synergistic enhancement in the levels of HLA-DRα and CD74 proteins, underscoring the intricate interplay of cytokine dynamics within cutaneous immunity [16].

The MHC II presentation pathway involves the pivotal role of the invariant chain (Ii or CD74) in guiding MHC II to endosomal–lysosomal compartments. This pathway facilitates the exchange of class II-associated invariant chain peptide (CLIP) for an antigenic peptide, a process assisted by HLA-DM [17]. When comparing our results with previous studies, it is crucial to highlight that disruptions in the endosomal–lysosomal pathway have been documented. This pathway plays a vital role in antigen processing and presentation, and its disruption can significantly alter immune response mechanisms [18]. Our temporal investigation into the colocalization of ovalbumin, serving as a model antigen, with early endosomal antigen 1 (EEA1) and lysosome-associated membrane protein 1 (LAMP1) markers reveals the dynamic processes that underlie antigen uptake and processing within keratinocytes. The cytokine-mediated rapid recruitment to endosomal and lysosomal pathways, particularly pronounced with IFN-γ treatment, initiates a strategic optimization of antigen processing mechanisms [19]. This acceleration is crucial, suggesting that cytokine exposure not only escalates the efficacy of antigen presentation but also refines the temporal efficiency of antigen processing, potentially modulating the qualitative and quantitative aspects of subsequent T-cell responses [20].

Furthermore, the extensive transcriptional profiling elucidates comprehensive reprogramming of keratinocyte functional paradigms in response to cytokine exposure. The pronounced alteration in gene expression, particularly in genes implicated in immune activation and antimicrobial defense, denotes a broad recalibration of keratinocyte functionality. This recalibration prepares the epidermis for enhanced microbial confrontations and possibly modulates the immunological milieu of the cutaneous environment [21].

A critical insight from this research is the clear delineation of how keratinocytes facilitate the modulation of T cell cytokine production. The increased production of IFN-γ, IL-2, IL-10, and IL-17A by CD4+ T cells following antigen presentation by cytokine-preconditioned keratinocytes highlights a significant interplay between the innate and adaptive immune systems. This modulation by keratinocytes underscores their role not just as structural components but as functional orchestrators in immune responses [22].

The pronounced cytokine release by naïve CD4+ lymphocytes in the presence of an antigen presented by treated keratinocytes suggests the involvement of both Th1 and Th2 pathways [23]. This could signify a shift towards a more balanced immune response, potentially aiding in the resolution of psoriatic inflammation or reflecting a return to homeostasis [24].

Building on these in vitro insights, the study expanded its research to include samples derived from psoriatic patients, stratifying participants into untreated (UT), topically treated (TT), and biologically treated (BT) cohorts. The cytokine secretion profiles in co-cultures from these groups offered a detailed perspective on how various psoriasis treatments influence the immune responses. In untreated psoriatic patients, the presence of OVA elicited a notable increase in cytokine production, indicating immunological activation consistent with the hyperreactive immune landscape observed in Ps. This finding aligns with our in vitro results, supporting the concept of a dysregulated immune response in Ps [25]. The comparison between TT and BT treatment groups after OVA stimulation revealed distinct differences in cytokine profiles. TT patients exhibited cytokine responses similar to those of untreated psoriatic patients (UT), with reductions in IFN-γ, IL-2, and IL-17F but a slight increase in IL-17A. Conversely, BT patients showed a distinct cytokine profile characterized by significantly reduced levels of IFN-γ, IL-2, IL-17F, and IL-22 compared to UT patients, highlighting the potent immune-modulating effects of biological treatments in attenuating pro-inflammatory pathways in psoriasis [26]. The experimental setup using naïve CD4+ lymphocytes from the same psoriatic patient groups further confirmed these findings, with a significant cytokine increase upon OVA stimulation observed across all groups. This indicates that the immune system’s responsiveness to antigenic stimuli is maintained across different treatment modalities, but the qualitative nature of this response may be altered by specific therapies.

Antigen presentation by keratinocytes via MHC class II (MHCII) is a critical element of the immune response, significantly influenced by the presence of IFN-γ. IFN-γ is a potent inducer of MHCII expression on various cell types, including keratinocytes. This leads to an increased expression of MHCII and associated invariant chain peptides, thereby enhancing the antigen-presenting capacity of keratinocytes [27]. IFN-γ has been shown to induce the expression of IRF1, a transcription factor that regulates MHCII expression, in epidermal keratinocytes. This highlights its role in enhancing the antigen-presenting ability of these cells [28]. Furthermore, IFN-γ is known to amplify pro-inflammatory gene expression in epidermal keratinocytes, underscoring its pivotal role in regulating immune responses in skin diseases such as Ps [29].

While ample data exist on the effects of IFN-γ, notably regarding its ability to induce MHCII expression and influence keratinocyte behavior, there is a relative scarcity of information on the broader spectrum of cytokines. IL-1β, IL-17A, and IL-22 are key cytokines produced during inflammatory responses, especially in Ps. They work together to enhance keratinocyte defense mechanisms, including the upregulation of antimicrobial peptides and pro-inflammatory cytokines. IL-1β, a potent pro-inflammatory cytokine, indirectly induces MHCII expression by promoting inflammation in various cell types, including keratinocytes [30]. This MHCII expression upregulation is part of the wider inflammatory response initiated by IL-1β, contributing to the antigen-presenting capacity of keratinocytes in inflammatory skin conditions. IL-17A and IL-2 primarily affect keratinocyte proliferation and barrier function. Although their direct impact on MHCII expression is less evident, their role in shaping the inflammatory milieu in the skin is thought to contribute to the overall antigen presentation process. The relevance of oncostatin M in skin inflammation is underscored by its potent modulatory effects on keratinocyte function, including the induction of members of the S100 family of proteins, which are involved in cutaneous inflammation [31]. The role of TNF-α in upregulating MHCII expression has also been established. For instance, one study showed that inhibiting the epidermal growth factor receptor augmented the expression of MHC class I and II genes in primary human keratinocytes, highlighting the potential of cytokine modulation to regulate MHC expression [32]. 

The complex interplay among IFN-γ, keratinocytes, and immune responses in skin disorders such as Ps represents a significant area of research interest. Our findings demonstrate that IFN-γ treatment markedly upregulates the expression of HLA-DRα and CD74 in keratinocytes, thus enhancing their antigen-presenting capabilities. This aligns with previous studies that emphasize the pivotal role of IFN-γ in modulating immune responses through the upregulation of MHC class II molecules on keratinocytes [7,33,34,35]. The secretion of cytokines by activated T cells interacting with IFN-γ-treated keratinocytes contributes to the inflammatory environment characteristics of Ps. Studies have highlighted the involvement of T cell-derived cytokines like IFN-γ, IL-17, and TNF-α in perpetuating the inflammatory cascade, suggesting potential therapeutic targets for modulating keratinocyte functionality [36]. The variability in cytokine profiles among psoriatic patients under different treatments reflects the personalized nature of immune responses in Ps, with insights into the modulation of cytokine production by biological treatments offering valuable information for targeted therapeutic approaches [37]. Keratinocytes dynamically modify their surface phenotype to interact with the environment, indicating their active involvement in modulating immune responses [38]. This adaptability is particularly evident in conditions like atopic dermatitis, where keratinocytes contribute to immune dysregulation and impact the disease’s pathophysiology through mechanisms of innate immunity [39]. The role of keratinocytes in the local activation of memory T cells further emphasizes their contribution to immune surveillance and response [40]. Upon activation, keratinocytes not only act as antigen-presenting cells but also secrete a range of cytokines, chemokines, and accessory molecules that modulate the function of immune cells, influencing the overall immune response in skin disorders [41]. Keratinocytes have been shown to initiate an antiviral defense program, showcasing their capacity to effectively counter viral infections [42]. Furthermore, their ability to induce rapid effector functions in antigen-specific memory CD4+ and CD8+ T cells highlights the crucial role keratinocytes play in initiating and modulating T cell responses [3]. Understanding the mechanisms through which keratinocytes process and present antigens via MHC class I and II molecules is essential for unraveling the immune landscape in skin disorders [43]. Additionally, keratinocytes can communicate with infiltrated immune cells through exosomes, potentially exacerbating skin inflammation in diseases like Ps [44]. In conclusion, the antigen presentation abilities of keratinocytes are central to the immune responses in skin disorders, shaping the interactions with immune cells and the modulation of cytokine expression. Their significance in the pathogenesis of various skin conditions underscores the need for further research into keratinocyte functions. 

While our study provides valuable insights into the immune-modulatory effects of IFN-γ and cytokines on keratinocytes and their implications for Ps, it is important to acknowledge some limitations. Firstly, the in vitro nature of the study may not capture the full complexity of in vivo immune responses, necessitating the need for further validation through animal models or clinical trials. Additionally, the focus on specific cytokine treatments and antigenic stimuli might limit the applicability of our findings to a wider range of immune responses in skin conditions.

In summary, our research elucidates the complex interplay between keratinocytes and T cells in the context of Ps [45]. The initial in vitro results lay the groundwork for understanding how keratinocytes mediate immune modulation, enriched by data derived from patients. Nonetheless, these outcomes should be interpreted with caution, recognizing the necessity of further investigations to confirm these effects in larger patient cohorts and through in vivo studies. Exploring the mechanisms behind the antigen presentation capabilities of keratinocytes, including the types of antigens they present and the conditions triggering their activation, will offer deeper insights into their role in skin immunity. Such investigations could lead to the identification of novel biomarkers for disease monitoring and progression, as well as new therapeutic targets. Our study enriches the existing body of knowledge on immune modulation in skin disorders and paves the way for future research, while refraining from making definitive statements regarding therapeutic efficacy at this juncture. An exemplary novelty of this scholarly endeavor is the dual-faceted methodology encompassing rigorous in vitro analyses complemented by empirical investigations using patient-derived specimens. This synergistic approach not only corroborates the initial laboratory-based insights but also imbues them with a pragmatic dimension, offering an elaborate perspective on the modulatory effects of various psoriatic treatments on cellular-level immune responses.

## 4. Materials and Methods

### 4.1. In Vitro Cell Culture

#### 4.1.1. Normal Human Keratinocytes (NHEKs) Culture

Primary Normal Human Keratinocytes isolated from the epidermis of four pooled Caucasian adult donors were sourced from PromoCell (Heidelberg, Germany), with cells at passage number 2–3 utilized for experiments. Cells were maintained in serum-free keratinocyte growth medium 2 (SFM, PromoCell, Heidelberg, Germany), supplemented with 0.004 mL/mL bovine pituitary extract, 0.125 ng/mL epidermal growth factor (recombinant human), 5 μg/mL insulin (recombinant human), 0.33 μg/mL hydrocortisone, 0.39 μg/mL epinephrine, 10 μg/mL transferrin (recombinant human), 0.06 mM CaCl_2_ (PromoCell, Heidelberg, Germany), and 1% antibiotic/antimycotic solution (A/A, Gibco, Thermo Fisher Scientific, Waltham, MA, USA), at 37 °C in a humidified atmosphere containing 5% CO_2_.

#### 4.1.2. Primary Psoriatic Keratinocytes (PHEKs) Isolation and Culture

Histologically psoriatic lesional skin tissues were assembled within the framework of our collaboration with the Department of Dermatology, Venereology, and Allergology at the Medical University of Gdansk, and signed informed consent was obtained from all subjects under protocols approved by the Independent BioEthics Committee of the Medical University of Gdansk (NKBBN/161/2017 and NKBBN/161-634/2018) and was conducted according to the principles of the Declaration of Helsinki. The 4 mm diameter skin biopsies were taken from the lesional skin (of buttocks) of 13 patients with psoriasis vulgaris. Primary psoriatic keratinocytes (PHEKs) were isolated from the lesional epidermis, separated by 10 U/mL Dispase II (Sigma-Aldrich, St. Louis, MO, USA) from the dermis and treated with 0.05% trypsin/EDTA (Gibco, Thermo Fisher Scientific, Waltham, MA, USA) to obtain a single cell suspension. The number of viable cells was determined using Trypan Blue staining (Sigma-Aldrich, St. Louis, MO, USA). Primary keratinocytes were seeded at a density of 5 × 10^6^ cells/cm^2^ in Dulbecco’s Modified Eagle Medium (DMEM, Gibco, Thermo Fisher Scientific, Waltham, MA, USA) supplemented with 10% fetal bovine serum (FBS, Gibco, Thermo Fisher Scientific, Waltham, MA, USA) and 1% antibiotic/antimycotic (A/A) solution (Gibco, Thermo Fisher Scientific, Waltham, MA, USA), at 37 °C, 5% CO_2_. After 24 h, the medium was replaced with SFM (PromoCell, Heidelberg, Germany), supplemented with 0.004 mL/mL bovine pituitary extract, 0.125 ng/mL epidermal growth factor (recombinant human), 5 μg/mL insulin (recombinant human), 0.33 μg/mL hydrocortisone, 0.39 μg/mL epinephrine, 10 μg/mL transferrin (recombinant human), 0.06 mM CaCl_2_ (PromoCell, Heidelberg, Germany), and 1% A/A solution (Gibco, Thermo Fisher Scientific, Waltham, MA, USA), and the cell cultures were maintained at 37 °C, 5% CO_2_.

#### 4.1.3. Isolation of Naïve CD4+ T Cells and CD4+ T Cells

##### Sample Collection and PBMC Isolation 

Human peripheral blood mononuclear cells (PBMCs) from 4 healthy donors were isolated from buffy coats using density gradient centrifugation. The material was obtained from the Regional Center for Blood Donation and Blood Treatment in Gdansk (Poland) (consent number: Dyr.M/073/03/AJC/2021). Briefly, buffy coats were diluted 1:1 with phosphate-buffered saline (PBS, pH 7.4, Biowest, Nuaillé, France) and carefully layered over Ficoll-Paque™ (Lymphosep, Biowest, Nuaillé, France). Centrifugation was performed at 400× *g* for 30–40 min at 20 °C without a break. The mononuclear cell layer (buffy coat) was collected, washed twice in PBS, and centrifuged at 300× *g* for 10 min at 21 °C. The cell pellet was resuspended in PBS for further analysis. Cell viability and purity were assessed using Trypan Blue staining (Sigma-Aldrich, St. Louis, MO, USA).

PBMCs from 13 psoriatic patients were collected under informed consent, following protocols approved by the Independent BioEthics Committee of the Medical University of Gdansk (NKBBN/161/2017 and NKBBN/161-634/2018) and were conducted according to the principles of the Declaration of Helsinki. Blood was drawn into BD Vacutainer^®^ CPT™ Mononuclear Cell Preparation Tubes (Becton Dickinson, Franklin Lakes, NJ, USA). The isolation of the PBMCs was performed using Ficoll-Paque™ density gradient centrifugation. Briefly, tubes were centrifuged at 1800× *g* for 30 min at 18–20 °C. The collected PBMCs were washed twice with PBS (Gibco, Thermo Fisher Scientific, Waltham, MA, USA) by centrifugation at 300× *g* for 10 min at 21 °C to remove the plasma, platelets, and Ficoll-Paque™. The supernatant was discarded after each wash. The final pellet was resuspended in RPMI 1640 medium (Biowest, Nuaillé, France) supplemented with 10% FBS (Biowest, Nuaillé, France) and 1% A/A solution (Biowest, Nuaillé, France). Cell count and viability were assessed using Trypan Blue staining (Sigma-Aldrich, St. Louis, MO, USA).

##### Magnetic Separation of Naïve CD4+ T Cells and CD4+ T Cells

The isolated PBMCs were subjected to a magnetic labeling and separation process aimed at enriching naïve CD4+ T Cells or CD4+ T cells through negative selection, utilizing a magnetic cell sorting system employing the MojoSort™ Human CD4 T Cell Isolation Kit and the MojoSort™ Human CD4 Naïve T Cell Isolation Kit, respectively, (Biolegend, San Diego, CA, USA) and QuadroMACS™ Separator (Miltenyi Biotec, Bergisch Gladbach, Germany), in accordance with the manufacturer’s protocol.

#### 4.1.4. Keratinocytes Stimulation with Cytokines

NHEKs were cultured in a SFM medium (PromoCell, Heidelberg, Germany) until they reached 70% confluency. To simulate psoriatic conditions, cells were divided into two groups: one representing the normal phenotype and the other the psoriatic phenotype. The psoriatic phenotype was induced by treating the cells with a cytokine mixture termed 5MIX, consisting of interleukin (IL)-1α, IL-17A, IL-22, oncostatin M (OsM), and tumor necrosis factor alpha (TNF-α) (Abcam, Cambridge, UK), each at 2 ng/mL for 24 h at 37 °C, 5% CO_2_. Additionally, both groups were treated with IFN-γ (Thermo Fisher Scientific, Waltham, MA, USA) at a concentration of 500 U/mL. PHEKs were stimulated only with 500U/mL IFN-γ for 24 h.

#### 4.1.5. Keratinocyte Stimulation with Ovalbumin

For antigen exposure experiments, NHEKs or PHEKs (stimulated as described in point Section 4.1.4) were incubated with 1 μg/mL ovalbumin (OVA, Sigma-Aldrich St. Louis, MO, USA) or 1 μg/mL Ovalbumin-Atto 594 conjugate (Sigma-Aldrich St. Louis, MO, USA) for immunofluorescence studies. The cells were exposed to OVA for 15, 30, 45, and 60 min (for co-localization studies) or for 24 h (for cytokines production studies) to assess both the immediate and prolonged effects of antigen stimulation.

#### 4.1.6. Co-Culture of NHEKs or PHEKs with Naïve CD4+ T Cells and CD4+ T Cells

Keratinocyte cell cultures were stimulated as described in points Section 4.1.4 and Section 4.1.5. Following treatment with cytokines and ovalbumin, the cells were meticulously washed five times with PBS to ensure the complete removal of any residual cytokines and ovalbumin from the culture environment. Subsequently, to the NHEK cultures, 1 × 10^6^/mL of either naïve CD4+ T cells or CD4+ T cells, which were isolated and pooled from 4 healthy donors, as described in Section 4.1.3, were added for 48 h. Analogously, keratinocytes isolated from the lesional skin of psoriatic patients were co-cultured with autologous naïve CD4+ T cells or CD4+ T cells for 48 h. 

### 4.2. Western Blotting

NHEKs (prepared as described in point Section 4.1.4) were lysed using RIPA lysis buffer (50 mM Tris-HCl, pH 7.4, 150 mM NaCl, 1% NP-40, 0.5% sodium deoxycholate, 0.1% SDS) supplemented with protease and phosphatase inhibitors (cOmplete Protease Inhibitor Cocktail and PhosSTOP, respectively; Roche, Penzberg, Germany), followed by quantification with the Pierce™ BCA Protein Assay Kit (Thermo Fisher Scientific, Waltham, MA, USA). Equal amounts of protein (30 μg) were denatured, loaded onto SDS-PAGE gels, and separated by electrophoresis. Separated proteins were transferred to polyvinylidene difluoride (PVDF) membranes (Sigma-Aldrich, St. Louis, MO, USA). Membranes were blocked with 5% Bovine Serum Albumin (BSA, Sigma-Aldrich, St. Louis, MO, USA) in Tris-buffered saline with 0.1% Tween^®^ 20 Detergent (TBST) to prevent non-specific binding. Membranes were incubated with primary antibodies: HLA-DR (1:500; sc-69673; Santa Cruz Biotechnology, Dallas, TX, USA), CD74 (1:500, sc-6262; Santa Cruz Biotechnology, Dallas, TX, USA), and β-actin (1:10,000; #4970; Cell Signaling Technology, Danvers, MA, USA) overnight at 4 °C, followed by HRP-conjugated secondary antibody, anti-mouse IgG (1:1000 for HLA-DR and CD74 and 1:10,000 for β-actin; #7076; Cell Signaling Technology, Danvers, MA, USA) for 2 h, room temperature, RT. Washes with TBST were performed after each antibody incubation. Protein bands were visualized using Pierce™ Reversible Protein Stain Kit for PVDF Membranes (Thermo Fisher Scientific, Waltham, MA, USA) and Azure 300^®^ Chemiluminescent Imager (Azure Biosystems, Dublin, CA, USA). Densitometry was quantified relative to a β-actin protein using Quantity One 1-D Analysis Software, v4.6.8 (Bio-Rad, Hercules, CA, USA) for normalization.

### 4.3. Fluorescence Microscopy

For immunostaining, NHEKs (prepared as described in point Section 4.1.4 and Section 4.1.5) were fixed with Image-iT™ Fixative Solution (Invitrogen, Waltham, MA, USA) for 10 min, permeabilized in 0.1% Triton X-100 (Sigma-Aldrich, St. Louis, MO, USA) for 30 min, blocked with 3% BSA in PBST for 1 h at 4 °C, and further incubated overnight at 4 °C with primary antibodies, Human Leukocyte Antigen – DRA isotype (HLA-DRA) mouse monoclonal antibody (1:500; sc-69673; Santa Cruz Biotechnology, Dallas, TX, USA), early endosome antigen 1 (EEA1) goat monoclonal antibody (1:500; ab206860, Abcam, Cambridge, UK), and lysosomal-associated membrane protein 1 (LAMP1) rabbit monoclonal antibody (1:500, #9091 Cell Signaling Technology, Danvers, MA, USA) in blocking buffer. The following day, cells were washed three times with PBS and incubated with fluorescently labeled secondary antibodies: goat anti-rabbit IgG H&L (1:1000; # A-11001 Alexa Fluor^®^ 488 Conjugate, Invitrogen, Waltham, MA, USA), donkey anti-goat IgG H&L (1:1000; ab150134, Alexa Fluor^®^ 555 Conjugate, Abcam, Cambridge, UK), and goat anti-rabbit IgG H&L (1:1000, #4412 Alexa Fluor^®^ 488 Conjugate, Cell Signaling Technology, Danvers, USA), and were then administered for 2 h at room temperature in the dark. The nuclei were stained with ProLong™ Gold Antifade Mountant with DNA Stain DAPI (Invitrogen, Waltham, MA, USA). The visualization was performed using a fluorescent microscope (DMI4000 B, Leica, Wetzlar, Germany) and LAS X Life Science Microscope Software, v1.4.7 28921 (Leica, Wetzlar, Germany) at a 100× magnification.

### 4.4. NanoString^®^ Analysis

Total RNA was extracted from NHEKs (stimulated as described in points Section 4.1.4 and Section 4.1.5) using the Total RNA Mini (A&A Biotechnology, Gdansk, Poland) according to the manufacturer’s instructions. RNA concentration and purity were assessed using NanoDrop Spectrophotometer (Thermo Fisher Scientific, Waltham, MA, USA). Gene expression profiling was performed using the NanoString nCounter^®^ Analysis System (NanoString^®^ Technologies, Seattle, WA, USA). A total of 40 ng of total RNA from each sample was hybridized to a pre-designed nCounter^®^ Host Response Panel according to the manufacturer’s protocol. Data acquisition was performed on the nCounter^®^ Digital Analyzer, capturing the number of times each target molecule was detected, directly correlating to the abundance of the target mRNA in the sample. Raw counts were normalized to the geometric mean of *ABCF1, ALAS1, GUSB, HPRT1, MRPS7, NMT1, NRDE2, OAZ1, PGK1, SDHA, STK11IP,* and *TBP* housekeeping genes to account for variability in RNA input, assay efficiency, and technical variation. Differential expression analysis was conducted using nSolver™ Analysis Software 4.0 with an advanced analysis module, R software, v4.3.3. Significance was set at a *p*-value < 0.05. Quality control measures included assessing the linearity and sensitivity of the assay across a range of RNA concentrations, as well as evaluating the reproducibility of technical replicates. Samples or probes not meeting quality control standards, such as those with low counts or high variability between replicates, were excluded from further analysis.

### 4.5. Measurment of Cytokine Levels Using Luminex^®^ xMAP^®^ Technology 

The protein level in the supernatant was determined using the Pierce™ BCA Protein Assay Kit and an equal amount of 10 µg of total protein/well was input per assay. Serial dilutions of cytokine standards were prepared to generate standard curves for each target cytokine. Assays performed on a MAGPIX^®^ instrument were based on Luminex^®^ xMAP^®^ technology (Luminex Corporation, Austin, TX, USA), using MILLIPLEX^®^ Human Cytokine/Chemokine/Growth Factor Panel A with IL-2, IL-4, IL-10, IL-17A, IL-17F, IL-22, and IFN-γ analytes. The data output gives the mean fluorescence intensity (mFI) as a measure of protein abundance. Data were analyzed using xPONENT software, v4.3 (Luminex Corporation, Austin, TX, USA), with cytokine concentrations calculated from the standard curves and expressed as pg/mL.

### 4.6. Statistics

Statistical analysis was performed with Prism 8.0.1.244 software (GraphPad, San Diego, CA, USA). Experimental data were presented as mean ± standard deviation. The normality of the data was determined using the Shapiro–Wilk test. The data distribution was then used to undertake statistical studies. Parametric analyses for normally distributed data included the use of two-way analysis of variance (ANOVA), followed by post-hoc testing to identify statistical differences. When normality assumptions were violated, non-parametric tests were used (Mann–Whitney U or Kruskal–Wallis test). *p*-values < 0.05 were considered significant.

## Figures and Tables

**Table 1:** Top 20 differentially expressed genes (DEGs) in an in vitro 2D model of Normal Human Epidermal Keratinocytes (NHEKs) treated with 5MIX: interleukin (IL)-1α, IL-17A, IL-22, oncostatin M (OsM), and tumor necrosis factor alpha (TNF-α), at 2 ng/mL each (A); interferon gamma (IFN-γ; 500 U/mL) (B); and the combination of 5MIX (2 ng/mL each) with IFN-γ (500 U/mL) (C); subjected to NanoString^®^ analysis using the nCounter^®^ Host Response gene expression panel. All experiments were conducted in biological triplicates (*n* = 3), and comparisons with *p* < 0.05 are displayed.

**A.**					
Gene Symbol	Log2 Fold Change	Std Error (log2)	Lower Confidence Limit (log2)	Upper Confidence Limit (log2)	*p*-Value
*DEFB103A/B*	2.23	0.448	1.36	3.11	0.00107
*ERN1*	0.58	0.145	0.295	0.865	0.00399
*NFATC4*	−0.485	0.138	−0.755	−0.215	0.00786
*GBP2*	2.89	0.841	1.24	4.54	0.00884
*TXN*	0.178	0.0562	0.0679	0.288	0.0132
*UBA52*	0.238	0.0804	0.0806	0.396	0.0181
*DDOST*	0.176	0.0631	0.0525	0.3	0.0235
*PSMB10*	0.507	0.182	0.15	0.863	0.0237
*IL1A*	0.412	0.155	0.108	0.716	0.0289
*LYN*	0.635	0.242	0.16	1.11	0.0305
*PLEKHA1*	0.123	0.0488	0.027	0.218	0.0361
*RAB7A*	0.232	0.0966	0.0432	0.422	0.0427
*MAFB*	0.716	0.304	0.12	1.31	0.0464
*IL13RA1*	0.546	0.233	0.0899	1	0.047
*IL1R2*	1.48	0.645	0.216	2.75	0.0509
*CEBPB*	0.173	0.0768	0.0227	0.324	0.0541
*ACVR1*	0.24	0.107	0.0314	0.449	0.0542
*LCP1*	0.33	0.15	0.0354	0.624	0.0594
*IL36B*	1.26	0.578	0.13	2.4	0.0605
*S100A12*	1.35	0.623	0.127	2.57	0.0625
**B.**					
Gene Symbol	Log2 Fold Change	Std Error (log2)	Lower Confidence Limit (log2)	Upper Confidence Limit (log2)	*p*-Value
*DEFB103A/B*	6.82	0.442	5.95	7.69	3.08 × 10^−7^
*PSMB10*	2.11	0.172	1.77	2.44	1.81 × 10^−6^
*GLA*	−1.2	0.104	−1.4	−0.996	2.82 × 10^−6^
*PRCP*	−1.01	0.0907	−1.19	−0.836	3.67 × 10^−6^
*DDAH2*	1.45	0.141	1.17	1.72	7.08 × 10^−6^
*GBA*	2.05	0.201	1.65	2.44	7.39 × 10^−6^
*LCN2*	2.54	0.251	2.05	3.03	7.82 × 10^−6^
*NFATC4*	−1.33	0.138	−1.6	−1.06	1.10 × 10^−5^
*IL32*	3.67	0.38	2.92	4.42	1.11 × 10^−5^
*CXCL9*	8.54	0.893	6.79	10.3	1.18 × 10^−5^
*OS9*	0.494	0.0519	0.392	0.595	1.22 × 10^−5^
*NOTCH1*	−1.31	0.138	−1.58	−1.04	1.23 × 10^−5^
*LDHB*	−1.32	0.14	−1.6	−1.05	1.32 × 10^−5^
*IL36B*	4.82	0.531	3.78	5.86	1.73 × 10^−5^
*PIK3CD*	−0.813	0.0909	−0.991	−0.635	1.94 × 10^−5^
*MAPK9*	−0.478	0.0535	−0.583	−0.373	1.97 × 10^−5^
*AKT1*	−0.427	0.0486	−0.523	−0.332	2.20 × 10^−5^
*CD59*	1.11	0.127	0.858	1.36	2.35 × 10^−5^
*IL33*	−4.18	0.512	−5.19	−3.18	3.73 × 10^−5^
*THOP1*	−1.03	0.126	−1.28	−0.783	3.77 × 10^−5^
**C.**					
Gene Symbol	Log2 Fold Change	std Error (log2)	Lower Confidence Limit (log2)	Upper Confidence Limit (log2)	*p*-Value
*DEFB103A/B*	5.22	0.442	4.35	6.08	2.44 × 10^−6^
*PSMB10*	1.68	0.173	1.34	2.02	1.08 × 10^−5^
*AKT1*	−0.465	0.0486	−0.56	−0.369	1.18 × 10^−5^
*CXCL9*	7.51	0.894	5.76	9.26	3.07 × 10^−5^
*DDAH2*	1.18	0.142	0.904	1.46	3.22 × 10^−5^
*LCN2*	2.04	0.251	1.54	2.53	3.96 × 10^−5^
*PRCP*	−0.672	0.0907	−0.85	−0.494	7.53 × 10^−5^
*NFATC4*	−1.02	0.138	−1.29	−0.747	7.76 × 10^−5^
*GBA*	1.43	0.201	1.04	1.83	9.89 × 10^−5^
*CD68*	1.28	0.187	0.913	1.65	0.000132
*DYSF*	1.3	0.193	0.925	1.68	0.000143
*RSAD2*	5.12	0.786	3.58	6.66	0.000184
*GLA*	−0.667	0.104	−0.87	−0.464	2.00 × 10^−4^
*RACK1*	−0.388	0.0607	−0.507	−0.269	0.000211
*PIK3CD*	−0.576	0.0909	−0.754	−0.398	0.000224
*OS9*	0.315	0.0519	0.213	0.416	3.00 × 10^−4^
*HLA-A*	0.836	0.138	0.564	1.11	0.000309
*IL32*	2.3	0.381	1.55	3.04	0.000311
*TXN*	0.338	0.0562	0.228	0.448	0.000319
*HLA-B*	0.972	0.163	0.652	1.29	0.000341

**Table 2:** Overview of treatments administered to psoriatic patients, including evaluation metrics such as the Psoriasis Area and Severity Index (PASI) for assessing the disease severity, Dermatology Life Quality Index (DLQI) for life quality impact, and Body Surface Area (BSA) for quantifying the affected skin area. The treatment categories include UT (Untreated), TT (Topical Treatment), and BT (Biological Treatment).

Patient Number	Type of Treatment	Medication Used	PASI	BSA	DLQI
1	UT	-	18.1	47	22
2	UT	-	13.2	28	4
3	UT	-	12.9	34	15
4	UT	-	22.7	34	18
5	UT	-	10.4	21	24
6	TT	clobetasol propionate	3	6	0
7	TT	clobetasol propionate	1.8	4	8
8	TT	clobetasol propionate	5	5	4
9	TT	clobetasol propionate	4.4	5	3
10	BT	adalimumab	13.5	32	8
11	BT	adalimumab	18.3	35	18
12	BT	ustekinumab	9.6	24	12
13	BT	ustekinumab	23.4	46	13

## Data Availability

Data supporting the reported results are not publicly available due to privacy or ethical restrictions. Specific details and raw data can be made available upon reasonable request to the corresponding author.
